# Serum Acylcarnitines Profile in Critically Ill Survivors According to Illness Severity and ICU Length of Stay: An Observational Study

**DOI:** 10.3390/nu15102392

**Published:** 2023-05-19

**Authors:** Anne-Françoise Rousseau, Alice Dongier, Camille Colson, Pauline Minguet, Jean-Olivier Defraigne, Grégory Minguet, Benoit Misset, François Boemer

**Affiliations:** 1 Intensive Care Department and Burn Centre, University Hospital of Liège, University of Liège, 4000 Liège, Belgium; alice.dongier@gmail.com (A.D.); camille.colson@chuliege.be (C.C.); pauline.minguet@chuliege.be (P.M.); benoit.misset@chuliege.be (B.M.); 2 GIGA-Research, GIGA-I3 Thematic Unit, Inflammation and Enhanced Rehabilitation Laboratory (Intensive Care), University of Liège, 4000 Liège, Belgium; gminguet@chuliege.be; 3 Cardiovascular Surgery Department, University Hospital of Liège, University of Liège, 4000 Liège, Belgium; jo.defraigne@chuliege.be; 4 Anesthesiology Department, University Hospital of Liège, University of Liège, 4000 Liège, Belgium; 5 Biochemical Genetics Lab, Department of Human Genetics, University Hospital of Liège, University of Liège, 4000 Liège, Belgium; f.boemer@chuliege.be

**Keywords:** carnitine, critical illness, survivors, mitochondrial dysfunction, cardiac surgery

## Abstract

The acylcarnitine (AC) profile has been shown to be altered in survivors of a prolonged stay in intensive care unit (ICU), with higher short-chain derivates compared to reference ranges. The present study aimed at describing the AC profile of patients surviving a short ICU stay versus patients surviving a >7-day multiple organ dysfunction. Patients discharged from ICU after an elective and non-complicated cardiac surgery (CS) were recruited. For each CS, one to two adults, matched for gender and age, were recruited among patients enrolled in our post-ICU follow-up program after an ICU stay ≥7 days (PS). In both groups, the AC profile was determined during the week following ICU discharge. A total of 50 CS patients (SAPS II 23 (18–27)) survived an ICU stay of 2 (2–3) days and were matched to 85 PS patients (SAPS II 36 (28–51), *p* < 0.001) who survived an ICU stay of 11 (8–15.5) days. No carnitine deficiency was observed in either group. Their total AC/C0 ratio was similar: 0.355 (0.268–0.415) and 0.358 (0.289–0.417), respectively (*p* = 0.391). A ratio >0.4 representing a disturbed mitochondrial metabolism was observed in 26/85 (30.6%) PS patients and in 15/50 (30%) CS patients (*p* > 0.999). The long-chain ACs were elevated in both groups, with a greater increase in the CS group. The short-chain ACs were higher in the PS group: 1.520 (1.178–1.974) vs. 1.185 (0.932–1.895) μmol/L (*p* < 0.001). The role of the AC profile as potential marker of catabolism and/or mitochondrial dysfunction during the critical illness trajectory should be further investigated.

## 1. Introduction

L-carnitine, the physiologically active isomer of carnitine, is involved in the transport of long-chain fatty acids from the cytoplasm to the mitochondria, and thus is an indirect actor of the beta-oxidation of fatty acids [1]. Carnitine also acts as a scavenger, binding acyl residues deriving from the intermediary metabolism of amino acids and facilitating their elimination. Both roles of carnitine result in its esterification into acylcarnitine derivatives. As such, the endogenous carnitine pool is comprised of L-carnitine and acylcarnitines (ACs), in which L-carnitine is the major representative, such that the normal acylcarnitine to L-carnitine ratio is 0.25 [2]. A ratio in the blood exceeding 0.4 is thought to represent disturbed mitochondrial metabolism [2,3].

In a recent study, we showed that the AC profile was altered after a prolonged stay in the ICU compared to a reference adult population [4]. Short-chain ACs were found in excess, while an increased AC/C0 ratio was observed in more than 25% of the cohort. These findings were thought to reflect catabolism and mitochondrial dysfunction. Muscle proteolysis in reaction to inflammation leads to muscle wasting: muscle loss occurs early after ICU admission, and more than 10% of the rectus femoris cross-sectional area is lost after the fifth day [5]. Muscle atrophy is a key contributor to ICU-acquired weakness, alongside functional and structural alterations in muscles and nerves [6]. These neuropathy and myopathy are thought to be underlined, at least partly, by a mitochondrial dysfunction [7]. Muscle atrophy and weakness increases are a frequent problem in ICU patients and survivors: their incidence is higher in the case of a prolonged ICU stay [8].

The AC profile in survivors of shorter ICU stays has never been investigated. It is unknown if an early response to different aggression could be translated in AC profile modifications. The aim of this observational study was to compare the AC profile of critically ill patients surviving a short ICU stay for a scheduled and uncomplicated cardiac surgery versus patients surviving a >7-day multiple organ dysfunction. We hypothesized that alterations in the AC profile (reflecting catabolism and mitochondrial dysfunction) would be more profound in long stayers.

## 2. Materials and Methods

This study was conducted in 2021 and 2022, after approval by the local Ethics Committee of our University Hospital (National Ref B7072021000006, Local Ref 2021/255, 14 September 2021). The participants were fully informed of the study purpose and procedure prior to enrollment. Our 6-unit adult intensive care department, admitting surgical and medical patients, is located in an academic hospital and includes 52 beds. 

### 2.1. Patients

A convenient sample of adults was recruited among patients consecutively scheduled for a cardiac surgery (CS) under sternotomy (coronary artery bypass graft (CABG), valve reconstruction or replacement, or a combined surgery). In our tertiary hospital, these patients are usually admitted to ICU during at least the first 48 h following surgery, aiming for anesthesia awakening, tracheal extubation, hemodynamic monitoring, and support. Exclusion criteria were an ICU length of stay (LOS) ≥48 h, known primary carnitine deficiency, and treated human immunodeficiency virus infection and ongoing treatment with valproate, cyclosporine, or cisplatin. Acylcarnitine profile was assessed during the 7 days following ICU discharge.

For each CS patient, 1 to 2 adults, matched for gender and age, were recruited among patients enrolled in our post-ICU follow-up program. For some years now, patients surviving a prolonged stay (PS) in ICU (defined as an ICU stay ≥7 days) are routinely invited to our post-intensive care follow-up, except in the cases of an end-of-life condition, coma, known dementia, or bedridden status. Additionally, they were not included if they were unable to communicate in French, the local language. The follow-up begins in the ward, during the first 7 days following ICU discharge: a nurse-led face-to-face standardized visit allows a first screening of mental and cognitive disorders using validated questionnaires. At that time, a blood analysis is also performed, focusing on inflammation, nutritional, and metabolic biomarkers. In this context, measurement of the acylcarnitine profile is part of our standard analysis. Patients were further excluded from the present study in the case of a treated HIV infection and ongoing treatment with valproate, cyclosporine, or cisplatin.

### 2.2. Serum Acylcarnitine Profiling

The biological data were generated from one single laboratory (Unilab, CHU de Liège) accredited according to ISO 15.189 guidelines.

In the text, Cx refers to the number of carbons in the acyl chain of carnitine derivatives (for instance, if x = 2, it refers to 2 carbons in the acyl chain), and the various classes of ACs are referred to as follows: (1) acetylcarnitine (C2) and short-chain ACs (SCACs: C3+C4+C5), (2) medium-chain ACs (MCACs: C6+C8+C10+C12), (3) long-chain ACs (LCACs: C14+C16+C18), (4) hydroxylated ACs (OH-ACs), and (5) dicarboxylic ACs (DC-ACs).

Blood samples were collected through a central or peripheral venous line placed for clinical use or through venous punction. Blood was drawn into a serum gel tube (BD Vacutainer, Becton, Dickinson and Company, Franklin Lakes, NJ, USA) before being centrifuged (3500 rpm, 15 min, 4 °C). Supernatant was frozen at −20 °C and stored for later analysis.

Serum AC concentrations (free carnitine (C0), C2-, C3-, C3DC-, C4-, C5-, C5:1-, C5DC-, C6, C6-DC-, C8-, C8:1-, C10-, C10:1-, C10:2-, C12-, C12:1-, C14-, C14:1-, C14:2-, C14-OH-, C16-, C16:1-, C16-OH-, C16-OH-, C18-, C18:1-, C18:2-, C18-OH-, C18:1-OH-, C18:2-OH-carnitine) were determined by flow injection on a TQ5500 tandem mass spectrometer (Sciex, Framingham, MA, USA), using MassChrom^®^ kit (Chromsystems, Munich, Germany) with slight modifications [9,10,11,12].

Reference ranges for all acylcarnitines were previously established in our same accredited lab, using the same flow injection—mass spectrometry method, according to EP28 CLSI (Clinical and Laboratory Standards Institute) guidelines. The reference intervals were defined using 50 serum samples of apparently healthy (and non-hospitalized) individuals aged from 18 to 81 years (55 [43–62.5] years), including 46% males (23/50). 

### 2.3. Other Collected Data 

Demographic data and data related to the ICU stay were collected retrospectively and extracted from the medical charts. 

Some ancillary biochemical parameters were also recorded. The amino acid profile was determined on the same blood samples by liquid chromatography coupled to mass spectrometry (LCMS) using aTRAQ assay (Sciex, Framingham, MA, USA) [13].

### 2.4. Analysis

Statistical analysis was performed using Graphpad Prism (version 9.0 for Mac OSX, Graphpad Inc., San Diego, CA, USA). Normality was assessed using the Shapiro–Wilk test. As some datasets did not pass the normality test, results were expressed as medians with lower and upper quartiles [Q1–Q3] for quantitative parameters. Qualitative variables were described using count and percent. Comparisons between data were made using the Mann–Whitney test and using Fisher’s exact test for categorical variables. A *p* value < 0.05 was considered statistically significant. 

## 3. Results

Between November 2021 and March 2022, 50 CS patients were analyzed after a CABG surgery (39/50, 78%), mitral or aortic valve replacement (7/50, 14%), or mixed surgery (4/50, 8%). Blood samples were obtained 4 [2–5] days after ICU discharge. They were matched to 85 PS patients: their AC profile was assessed 6 [4–8] days after ICU discharge. Descriptive characteristics of the included subjects in both groups are detailed in Table 1.

The AC profiles of the two groups of ICU survivors, compared to the reference ranges, are detailed in Table 2. The two profiles were different, mainly in terms of short-chain and long-chain ACs. The sums of SCACs and LCACs in the two groups are reported in the Table 3. 

On the contrary, C0 (free carnitine) concentration was similar in both groups (Table 2). No patients in either of the two groups presented with a C0 deficiency (i.e., C0 concentration < percentile 2.5 of the reference population). 

The AC/C0 ratio was similar in the two groups: 0.358 [0.289–0.417] in CS patients and 0.355 [0.268–0.415] (*p* = 0.391). A ratio >0.4 was observed in a similar proportion of patients in the two groups: 15/50 (30%) patients in CS group and 26/85 (30.6%) patients in the PS group, respectively (*p* > 0.999). In the PS group, the SAPS II at admission was similar in patients with a normal AC/C0 ratio compared to those with an abnormal AC/C0 ratio: 37 [28–49.5] and 33 [28.5–54.5], respectively (*p* = 0.891).

Ancillary blood biomarkers measured at the same time as ACs are described in Table 4. The three branched-chain amino acids’ (leucine, isoleucine, valine) concentrations were significantly higher in the CS group compared to the PS group (*p* < 0.001).

## 4. Discussion

This study is the first to describe and compare the AC profile after a prolonged versus a shorter stay in ICU. Measurement of the AC profile was performed using a reference method (mass spectrometry), allowing the measurement of C0 and the entire range of its acyl-esters. We confirmed that carnitine deficiency was anecdotal, even after a prolonged critical illness. However, the AC profile was quite different between the two categories of ICU survivors. 

The main finding was related to short-chain ACs: their blood levels were lower after a short ICU stay compared to a prolonged stay. In these later survivors, observed SCAC levels were consistent with the levels described in our previous published study comparing ICU survivors to a reference population [4]. In the present study, these findings were associated with a significant difference in BCAA concentrations: leucine, isoleucine, and valine concentrations were lower after a prolonged stay compared to a shorter stay. Accumulation of ACs, known as by-products of the mitochondrial fatty acid oxidation, can also result from the degradation of BCAA. The catabolism of BCAA generates acyl-CoA derivates, which will transfer their acyl group to carnitine to form mainly C3 and C5 ACs [14]. SCACs are thus considered as a marker of protein catabolism. BCAA serves as an important energy substrate in muscles during periods of stress [15]. An acute major injury such as trauma or sepsis induces an accelerated metabolic rate, a net negative protein balance in muscle, and a depletion of the body’s protein store [16]. It would thus make sense to hypothesize that a major surgery would lead to a lesser catabolic response. That is obviously what we observed in the present study with the levels of SCACs. That said, SCAC levels could be an interesting marker of the catabolism during a critical illness trajectory, either in terms of the extent of the catabolic response or in terms of the transition between catabolic and anabolic profiles during the recovery phase. This requires further investigation. The clinical need for such a biomarker is burning: there is currently no tool helping clinicians to adapt energy and protein targets according to the metabolic changes that are supposed to occur after a critical injury [17]. 

An increased AC/C0 ratio was observed in one-third of the survivors of a prolonged stay, a little bit more than in our previously studied cohort [4]. However, the same proportion was also observed in survivors of a shorter ICU stay. An AC/C0 ratio in the blood exceeding 0.4 is thought to reflect disturbed mitochondrial metabolism [2,3]. Mitochondrial dysfunction is observed as early as the acute phase of a critical illness, at least in the muscles [18]. Inflammation is a well-known trigger for mitochondrial dysfunction [19,20]. Residual or persistent inflammation is typically observed early after ICU discharge [4]. Skeletal muscle, rich in mitochondria, is vulnerable to the various forms of mitochondrial alterations observed early after critical illness, thus explaining at least partly the neuromuscular failure in ICU survivors [21]. According to our present observation, mitochondrial dysfunction, based on the AC/C0 ratio, was observed independently of the initial insult severity or ICU LOS. Investigating the mitochondrial function is not easy in clinical practice, and the AC/C0 ratio could be a non-invasive and simple method to detect it, if further proved against a reference or other validated methods, such as skin or muscle biopsy or proteomic analysis [22]. 

In the present study, LCACs were elevated in both groups compared to the reference population [4]. LCACs are also considered as markers of mitochondrial dysfunction, as increased levels are associated with the impaired mitochondrial ability of fatty acid oxidation [3]. Such signature has already been observed in sepsis [23,24], associated with an increased mortality. During the COVID-19 pandemic, elevation of LCACs was observed in the post-acute phase of a moderate infection [25] or in long-COVID patients [26]. Lower fatty acid oxidation in mitochondria has thus been suspected to explain the observed exercise intolerance in these patients [27]. 

Unlike we anticipated, LCACs were higher in the patients of the CS group, i.e., the patients with the less severe insult and the shorter ICU stay. This group consisted of patients scheduled for cardiac surgery. In previous publications, the accumulation of circulating LCACs has been associated with heart failure or coronary artery disease, proportionally to the disease severity [28]. In cardiovascular diseases, LCACs are markers of a mitochondrial dysfunction triggered by the known cardiovascular risk factors. However, LCACs also contribute to disease severity, with direct effects on electrophysiology and cardiac contractility. This could explain the present findings.

Some limitations need to be acknowledged. First, the number of included patients is relatively small. No sample size has been calculated in absence of previously published relevant data. However, the PS group has been matched for age and sex with the CS group, limiting some bias. Second, this study is a retrospective analysis of prospectively recorded data. It would have been interesting to concomitantly investigate functional outcomes such as muscle mass or strength. Unfortunately, such data were not available for the present cohorts. Third, this is a cross-sectional study without longitudinal assessment of the AC profile. Further studies should analyze repeated AC profile assessments all along the trajectory of the patients, from ICU admission to long-term follow-up. This work is ongoing in our follow-up clinic. Finally, it would have been informative to have pre-ICU values. Such evaluation is, by definition, challenging as more than half of ICU admissions are unexpected. 

## 5. Conclusions

In ICU survivors discharged after a prolonged stay for a severe critical illness, higher short-chain ACs characterized a substantial BCAA depletion and catabolic status, compared to survivors of a shorter ICU stay. Both groups showed biological signs of mitochondrial dysfunction: elevated long-chain ACs and an abnormal AC/C0 ratio were observed in one-third of the patients, independently of illness severity. Further investigations should specify the potential role of the AC profile as a marker of catabolism and/or mitochondrial dysfunction during the critical illness trajectory.

## Figures and Tables

**Table 1:** Demographics in the two groups.

Data			Survivors of a Cardiac Surgery (CS Group) n = 50	Survivors of a Prolonged Stay in ICU (PS Group) n = 85	*p* Value
Age, y			70.9 [65–77.6]	68 [63–73]	0.0942
Male, n (%)			40 (80)	67 (78.8)	>0.999
Weight, kg			84 [72.7–94]	77.7 [68.2–87.8]	0.081
BMI, kg/m^2^			28.7 [26.1–30.5]	26.6 [23.5–29.4]	0.028
Comorbidities, n (%)	Cardiovascular ^a^		50 (100)	68 (80)	<0.001
	Respiratory ^b^		14 (28)	30 (35.3)	0.449
	Neurological ^c^		10 (20)	8 (9.4)	0.114
	Chronic kidney disease		11 (22)	14 (16.5)	0.493
	Diabetes		13 (26)	24 (28.2)	0.843
	Cirrnhosis		0	2 (2.3)	0.530
	Immunosuppression		0	9 (10.6)	0.026
	Cancer		5 (10)	23 (27.1)	0.027
Admission category, n (%)	Medical		-	42 (49.4)	-
	Surgical		50 (100)	43 (50.6)	-
Primary failure, n (%)	Cardiovascular		50 (100)	49 (57.7)	-
	Pulmonary		-	21 (24.7)	-
	Neurologic		-	2 (2.4)	-
	Digestive and hepatic		-	6 (7)	-
	Polytrauma		-	1 (1.2)	-
	Other		-	6 (7)	-
SAPS II			23 [18–26.7]	40.5 [29–54.5]	<0.001
Mechanical ventilation > 24 h, n (%)			0	56 (65.9)	-
Duration of mechanical ventilation, d			-	3 [1–11.5]	-
Renal replacement therapy, n (%)			-	2 (2.3)	-
Duration of renal replacement therapy, d			-	5 and 8 days	-
Extracorporeal membrane oxygenation, n (%)			-	2 (2.3)	-
Propofol-based sedation, n (%)			-	58 (68.2)	-
Duration of propofol infusion, d			-	2.5 [1–8.2]	-
Type of nutrition during ICU stay, n (%)		Oral nutrition	50 (100)	56 (65.9)	-
		Enteral nutrition	-	33 (38.8)	-
		Parenteral nutrition	-	12 (14.1)	-
		None	-	9 (10.6)	-
ICU LOS, d			2 [2,3]	11 [8–15.5]	<0.001

Data are expressed as medians with lower and upper quartiles [Q1–Q3]. BMI: body mass index; ICU: intensive care unit, LOS: length of stay; SAPS II: Simplified Acute Physiology Score II. ^a^ Ischemic heart disease, valvular disease, cardiomyopathies, chronic heart disease, hypertension, pulmonary embolism. ^b^ Asthma, chronic obstructive pulmonary disease, and interstitial lung diseases. ^c^ Cerebral vascular accident, acute encephalitis, Parkinson’s disease.

**Table 2:** Acylcarnitines concentration in the two groups.

Acylcarnitines (μmol/L)	Reference Range	CS Group n = 50	PS Group n = 85	*p* Value
C0	14.95–84.34	45.58 [39.2–55.75]	50.79 [38.22–62.93]	0.072
C2	2.71–21.28	12.87 [10.61–14.54]	12.34 [9.33–17.53]	0.945
C3	0.086–3.329	0.470 [0.337–0.593]	0.670 [0.448–0.917]	<0.001
C3-DC	0.007–0.221	0.090 [0.067–0.130]	0.090 [0.049–0.135]	0.340
C4	0.038–0.400	0.200 [0.147–0.270]	0.300 [0.198–0.420]	<0.001
C5	0.024–0.202	0.110 [0.080–0.150]	0.110 [0.080–0.150]	0.816
C5:1	0.004–0.043	0.020 [0.020–0.030]	0.020 [0.011–0.036]	0.168
C5-OH	0.011–0.073	0.050 [0.047–0.062]	0.068 [0.050–0.081]	0.017
C5-DC	0.021–0.267	0.200 [0.157–0.270]	0.203 [0.127–0.290]	0.058
C6	0.011–0.164	0.090 [0.060–0.140]	0.090 [0.060–0.160]	0.289
C6-DC	0.019–0.578	0.040 [0.030–0.060]	0.070 [0.040–0.145]	0.005
C8	0.016–0.291	0.145 [0.087–0.220]	0.120 [0.080–0.173]	0.233
C8:1	0.019–0.331	0.130 [0.090–0.200]	0.160 [0.118–0.255]	0.01
C10	0.023–0.622	0.200 [0.130–0.303]	0.159 [0.100– 0.234]	0.032
C10:1	0.016–0.265	0.100 [0.080–0.140]	0.090 [0.060–0.133]	0.196
C10:2	0.004–0.050	0.020 [0.020–0.030]	0.020 [0.012–0.030]	0.651
C12	0.011–0.239	0.090 [0.057–0.120]	0.060 [0.040–0.090]	0.003
C12:1	0.012–0.253	0.085 [0.060–0.123]	0.076 [0.050–0.110]	0.552
C14	0.008–0.081	0.060 [0.040–0.080]	0.040 [0.032–0.057]	<0.001
C14:1	0.016–0.315	0.120 [0.090–0.153]	0.083 [0.060–0.121]	<0.001
C14:2	0.005- 0.080	0.040 [0.030–0.060]	0.030 [0.020–0.041]	<0.001
C14-OH	0.002–0.016	0.020 [0.010–0.020]	0.010 [0.009–0.020]	0.005
C16	0.060–0.293	0.270 [0.200–0.380]	0.190 [0.140–0.290]	<0.001
C16:1	0.007–0.154	0.050 [0.047–0.070]	0.041 [0.030–0.060]	0.001
C16-OH	0.001–0.009	0.010 [0.010–0.020]	0.010 [0.005–0.020]	0.032
C18	0.019–0.082	0.100 [0.087–0.125]	0.070 [0.045–0.110]	<0.001
C18:1	0.048–0.479	0.270 [0.237–0.320]	0.220 [0.162–0.290]	0.001
C18:2	0.012–0.106	0.080 [0.070–0.090]	0.063 [0.050–0.100]	0.018
C18:1-OH	0.001–0.010	0.020 [0.010–0.030]	0.010 [0.004–0.020]	<0.001
C18:2-OH	0.001–0.006	0.010 [0.001–0.020]	0.010 [0.002–0.010]	<0.001

Data are expressed as medians with lower and upper quartiles [Q1–Q3].

**Table 3:** Classes of acylcarnitines in the two groups.

Acylcarnitines (μmol/L)	Reference Range	CS Group n = 50	PS Group n = 85	*p* Value
SCACs	0.270–4.071	1.185 [0.932–1.895]	1.520 [1.178–1.974]	0.010
LCACs	0.195–1.295	1.090 [0.935–1.293]	0.830 [0.660–1.105]	<0.001

Data are expressed as medians with lower and upper quartiles [Q1–Q3]. SCACs: short-chain ACs; LCACs: long-chain ACs.

**Table 4:** Ancillary biochemical parameters.

Biomarkers	Reference Range	CS Group n = 50	PS Group n = 85	*p* Value
Leucine (μmol/L)	73.5–228	155.5 [134.8–196.3]	127 [92.1–147.5]	<0.001
Isoleucine (μmol/L)	36.5–132	97.5 [73.4–116.8]	75 [55.8–94.85]	0.001
Valine (μmol/L)	105–352	259 [203–306]	201 [170–239]	<0.001

Data are expressed as medians with lower and upper quartiles [Q1–Q3].

## Data Availability

The datasets used and/or analyzed during the current study are available from the corresponding author on reasonable request.
